# Prospective observational study on tracheal tube cuff pressures in emergency patients– is neglecting the problem the problem?

**DOI:** 10.1186/1757-7241-21-83

**Published:** 2013-12-04

**Authors:** Falko Harm, Mathias Zuercher, Marco Bassi, Wolfgang Ummenhofer

**Affiliations:** 1 Department for Anesthesia, Surgical Intensive Care, Prehospital Emergency Medicine and Pain Therapy, University Hospital Basel, Basel Switzerland; 2 Department of Anaesthesia, St. Claraspital, Basel, Switzerland

**Keywords:** Tube cuff pressure, Airway management, Tracheal intubation, Emergency medicine, Aeromedical transport, Prehospital monitoring, Patient safety

## Abstract

**Background:**

Inappropriately cuffed tracheal tubes can lead to inadequate ventilation or silent aspiration, or to serious tracheal damage. Cuff pressures are of particular importance during aeromedical transport as they increase due to decreased atmospheric pressure at flight level. We hypothesised, that cuff pressures are frequently too high in emergency and critically ill patients but are dependent on providers’ professional background.

**Methods:**

Tracheal cuff pressures in patients intubated before arrival of a helicopter-based rescue team were prospectively recorded during a 12-month period. Information about the method used for initial cuff pressure assessment, profession of provider and time since intubation was collected by interview during patient handover. Indications for helicopter missions were either Intensive Care Unit (ICU) transports or emergency transfers. ICU transports were between ICUs of two hospitals. Emergency transfers were either evacuation from the scene or transfer from an emergency department to a higher facility.

**Results:**

This study included 101 patients scheduled for aeromedical transport. Median cuff pressure measured at handover was 45 (25.0/80.0) cmH_2_O; range, 8-120 cmH_2_O. There was no difference between patient characteristics and tracheal tube-size or whether anaesthesia personnel or non-anaesthesia personnel inflated the cuff (30 (24.8/70.0) cmH_2_O vs. 50 (28.0/90.0) cmH_2_O); p = 0.113.

With regard to mission type (63 patients underwent an emergency transfer, 38 patients an ICU transport), median cuff pressure was different: 58 (30.0/100.0) cmH_2_O in emergency transfers vs. 30 (20.0/45.8) cmH_2_O in inter-ICU transports; p < 0.001. For cuff pressure assessment by the intubating team, a manometer had been applied in 2 of 59 emergency transfers and in 20 of 34 inter-ICU transports (method was unknown for 4 cases each). If a manometer was used, median cuff pressure was 27 (20.0/30.0) cmH_2_O, if not 70 (47.3/102.8) cmH_2_O; p < 0.001.

**Conclusions:**

Cuff pressures in the pre-hospital setting and in intensive care units are often too high. Interestingly, there is no significant difference between non-anaesthesia and anaesthesia personnel. Acceptable cuff pressures are best achieved when a cuff pressure manometer has been used. This method seems to be the only feasible one and is recommended for general use.

## Background

For emergency patients, tracheal intubation with a cuffed tube remains the gold standard for securing the airway. Compared with other airway tools, only cuffed tracheal tubes are able to prevent aspiration. In addition, for controlled ventilation, air leakage must be minimised. This is especially important during the transfer and relocation of patients. In emergency situations, the risk of over-inflation is high [1-3]. There is evidence from studies and case reports that over-inflation of the tracheal cuff can cause various complications including rather benign complaints such as sore throat or hoarseness and mild mucosal damage expressed by transient blood-streaked expectorant [4-6], but also serious complications such as tracheal stenosis or even tracheoesophageal fistula or tracheal rupture [7-12].

Possible damage from inappropriately high cuff pressures is a well-known hazard in long-term (days) ventilated patients but is a less recognised problem in short-term (hours) intubated patients. Nevertheless, a recent publication demonstrated that mucosal damage also occurs in short-term (1-3 hours) intubated patients [4]. Emergency patients are especially prone to cuff pressures that remain unmeasured for hours [1].

In addition, cardiac output and blood pressure in emergency patients are often compromised, and low capillary pressure has been shown to influence tracheal mucosal perfusion [13,14] resulting in damage that can occur at lower cuff pressures than in haemodynamically stable patients.

Because situations in emergency and critically ill patients are more complex and team compositions are often not as well-rehearsed as in elective hospital intubation settings, we hypothesised that cuff pressures are frequently too high and depend on the provider’s professional background.

## Methods

The study was approved by the local Institutional Review Board (Ethikkommission beider Basel) as a quality assurance investigation. Requirement for written informed consent was waived by the Institutional Review Board.

### Study design

This prospective observational study occurred over a 12-month period. Inclusion criteria included all mechanically ventilated patients scheduled for aeromedical transport and intubated with a cuffed tracheal tube. Exclusion criteria were: (i) patients who were intubated by the study group; (ii) devices other than regular tracheal tubes (e.g. double-lumen tubes, laryngeal tubes or tracheostomy tubes); (iii) technical problems (e.g. incompatibilities between the cuff pilot and the cuff-pressure manometer or damaged tube cuffs); and (iv) patients in whom the study setting seemed unsuitable due to safety concerns (e.g. heavy workload for the emergency physician, cardiovascular or respiratory instability, multi-resistant infections, or time pressure due to surrounding circumstances such as weather or daylight conditions).

Initial cuff pressures were measured before loading the patient into the helicopter, using a commercially available cuff-pressure manometer (Mallinckrodt, Hazelwood, MO, USA). As all emergency physicians working on the helicopter were anaesthesiologists familiar with the manometer from their anaesthesia practice, no formal training was required. Cuff pressure was considered “elevated” if the value was ≥30 cmH_2_O.

The local team present at the handover was questioned about the professional background of the person who had inflated the cuff (anaesthesia vs. non-anaesthesia personnel and physician vs. nurse/paramedic staff). In addition, the technique used for cuff inflation was recorded, specifically if cuff pressure was measured or if it was estimated clinically.

For safety reasons, after measurement by the investigating helicopter team, cuff pressure was adjusted to 25 cmH_2_O. Besides this intervention, there was no further change in patient management.

### Setting

The study was performed in cooperation with Swiss Air-Rescue (REGA, Zurich, Switzerland). The helicopter base was located at the Euro-Airport Basel and serves the tri-border region of Switzerland, Germany and France. The helicopter emergency medical services crew consisted of a pilot, a paramedic-flight assistant and a board certified anaesthesiologist or anaesthesiologist at certification level with an additional board-certification in pre-hospital emergency medicine.

### Data collection and processing

The following parameters were assessed: cuff pressure before helicopter take off; type of mission (“emergency transfers” (patients with tracheal intubation on scene or in the emergency department) or “ICU transfers” (intensive care inter-hospital transfer)); time between intubation and the initial cuff pressure measurement; tube size and insertion depth (tip to teeth in cm); and patient characteristics (sex, age and estimated height and weight).

Data were recorded using printed forms and later transferred into a Microsoft Excel® 2000 worksheet (Microsoft Corp., Redmond, WA, USA).

### Data analysis

Data were analysed using IBM SPSS Statistics 19.0 (IBM Corp., Armonk, NY, USA). Data are presented as descriptive statistics (median (25/75 percentiles); mean ± SD). Mann-Whitney U test was used for comparison between continuous variables. Chi-square tests were used for categorical data. Values of p < 0.05 were considered significant.

## Results

**Figure 1:**
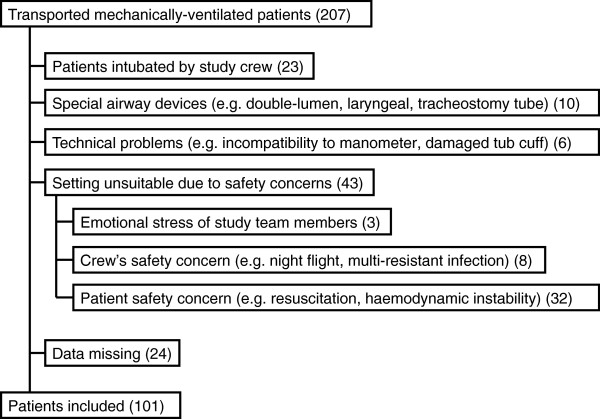
Selection of patients and exclusion criteria.

After exclusion based on the specified criteria, data of 101 patients were included in the study (Figure 1). Patient demographics were distributed as follows: 77 (76%) were male; mean age was 54 ± 20.1 years (range, 10-85 years), 95 (94%) were ≥18 years. Tube diameters are shown in Table 1. Regarding mission type, the majority of patients (n = 63) underwent an emergency transfer and 38 patients an ICU transfer.

**Table 1:** Absolute (relative) frequency of tracheal tubes used in women/men <18 years (n = 95)

	**TT 6.5**	**TT 7.0**	**TT 7.5**	**TT 8.0**	**TT 8.5**	**Σ**
**Female**	1 (4%)	5 (22%)	10 (44%)	7 (30%)	-	23 (100%)
**Male**	-	2 (3%)	10 (15%)	41 (59%)	16 (23%)	69 (100%)

TT = tracheal tube (internal diameter in mm). TT not recorded: n = 3. Patients <18 years: n = 6.

Median initial cuff pressure measured was 45 (25.0/80.0) cmH_2_O (mean 55 ± 34.7 cmH_2_O; range, 8-120 cmH_2_O). There was no difference in cuff pressures dependent on patient characteristics (i.e. sex, age) or tracheal tube diameter. Values of initial cuff pressure were not different if anaesthesia personnel (nurse or anaesthesiologist) or non-anaesthesia personnel (nurse or physician) had inflated the cuffs (median 30 (24.8/70.0) vs. 50 (28.0/90.0) cmH_2_O, mean 46 ± 31.5 vs. 60 ± 35.1 cmH_2_O); p = 0.113.

Mission type significantly influenced cuff pressure values: 58 (30.0/100.0) cmH_2_O for emergency transfers vs. 30 (20.0/45.8) cmH_2_O for ICU transfers (mean 64 ± 36.4 vs. 38 ± 24.5 cmH_2_O); p < 0.001.

Duration between tracheal intubation and patient handover was 40 ± 41.1 minutes (range, 0-220 minutes) in emergency transfers and 97 ± 167.4 minutes (range, 0-840 minutes) in ICU transfers. The highest percentage of elevated cuff pressures (defined as ≥30 cmH_2_O) was found in patients intubated within one hour of measuring (Table 2). Median cuff pressures in patients intubated <60 minutes (n = 65) and ≥60 minutes (n = 30) before measurement were 45 (26.5/87.5) and 42 (23.0/80.0) cmH_2_O (mean 56 ± 35.7 and 51 ± 35.4 cmH_2_O), respectively.

**Table 2:** Initial cuff pressures dependent on time intubated prior to the measurement

	**<60 minutes**	**60-119 minutes**	**≥120 minutes**
**n**	65	16	14
**P**_**init**_ **< 30**	19 (29%)	6 (38%)	5 (36%)
**P**_**init**_ **≥ 30**	46 (71%)	10 (62%)	9 (64%)

n = number of cases. P_init_ = initial cuff pressure (measured in cmH_2_O). Data missing: n = 6.

Different methods for cuff pressure assessment, if any, were applied by the intubating team: a manometer was applied in 22 cases (22%); a specific volume of air was inflated in 14 cases (14%); the palpatory method was used in 10 cases (10%); and the acoustic method was used in one case. In 46 patients (46%), cuff pressures had not been checked by any method, and in 8 cases the referring person could not provide any information on the type of measurement, if any. ICU transfers showed a higher percentage of “measurement” (20 of 34, 59%) compared with emergency transfers (2 of 59, 3%), for 4 patients in each group the technique was unknown.

**Figure 2:**
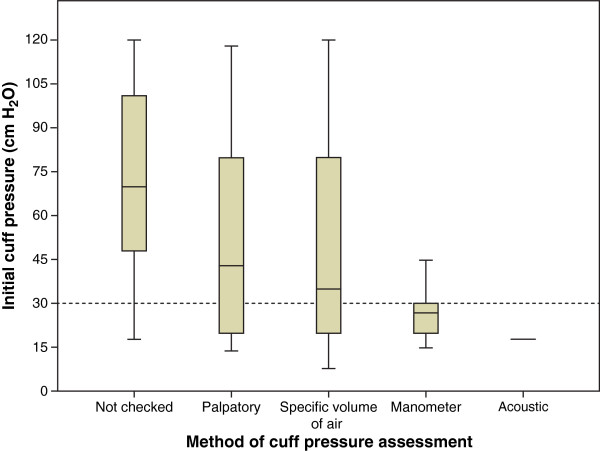
**Cuff pressure according to method of assessment.** Values are shown as median (25/75 percentiles) in cmH_2_O (n = number of cases). Cuff pressure not checked: 70 (47.3/102.8) cmH_2_O (n = 46); palpatory method: 43 (20.0/86.5) cmH_2_O (n = 10); specific volume of air: 35 (17.5/85.0) cmH_2_O (n = 14); cuff pressure manometer: 27 (20.0/30.0) cmH_2_O (n = 22); acoustic method: 18 cmH_2_O (n = 1). Dashed line indicates the upper safe cuff pressure limit. p < 0.001 for comparison of *checked by manometer* vs. *not checked*.

Different methods of checking showed different cuff pressure values (Figure 2). If a manometer was used, median cuff pressure was 27 (20.0/30.0) cmH_2_O, if not 70 (47.3/102.8) cmH_2_O (mean 27 ± 8.7 vs. 65 ± 35.6 cmH_2_O); p < 0.001. Apart from one cuff pressure evaluated using the acoustic method, manometer measurement was the only method with a median cuff pressure <30 cmH_2_O.

## Discussion

Tracheal tube cuff pressures in patients intubated prior to aeromedical transport are generally too high. This finding is independent of patient characteristics, tube diameter and time since intubation. In particular, professional background of those who performed intubation had no influence. Contrary to expectation, anaesthesia personnel did not reliably control the appropriateness of the cuff inflation. Cuff pressures were much higher in emergency transfers than in ICU transfers. Elevated cuff pressures in tracheally intubated patients prior to emergency transfer have been demonstrated in the prehospital and the emergency department setting [1-3,15,16]. Correlation between high cuff pressure and tracheal injury has been known for decades [13,17,18]. This may be especially harmful for intubated patients scheduled for airborne transportation. During helicopter transfer of tracheally intubated patients, cuff pressures have been shown to dangerously increase in spite of cuff control prior to ascent [19,20]. Similar results have been reported for fixed-wing air-medical retrieval [15,20] and in an *in vitro* study simulating flight-level changes in an altitude chamber [21]. If not controlled prior to or monitored during air transfer, high pre-existing pressures will reach extreme values.

Tracheal mucosal blood flow has been shown to be impaired at cuff pressures ≥30 cmH_2_O [13]. Animal data suggest ischaemic injury to the tracheal mucosa when cuff-to-tracheal wall pressures ≥20 mmHg persist for 15 minutes [22]. As many emergency transfers of patients involve haemodynamically unstable patients with significant hypotension, blood flow in the tracheal mucosa may be compromised at even lower inflation pressures. In a canine model, capillary mucosal perfusion was already significantly reduced at 22 mmHg mucosal contact pressure during hypotension (defined as a mean arterial pressure (MAP) of 50 mmHg during a period of 15 minutes) [14]. In a rabbit model, tracheal mucosal blood flow was diminished according to the ratio of cuff / MAP: if cuff pressure was 20 to 30% of MAP (which at a MAP of 75 mmHg, corresponds to cuff pressures of 15–22.5 mmHg), there already was a marked reduction of microvascular tracheal mucosal blood flow over the cartilages. To safely avoid total ischaemia, cuff pressure must not have exceeded 40% of MAP [20].

A recent multicentre study recorded cuff pressures in an operating room setting and showed much higher than optimal values if pressure was estimated by clinical judgement alone [4]. In addition, the same study demonstrated by clinical evaluation and fibreoptic control that sore throat, hoarseness and blood-streaked expectoration 24 hours after extubation and, respectively, mucosal injury observed by fibreoptic bronchoscopy immediately after removal of the tube were significantly higher in the non-pressure controlled group. These complications already occurred in procedures as short as 1-3 hours.

For brief procedures lasting only a few hours, most anaesthesiologists pay little attention to tube cuff inflation pressure [4]. Several methods to prevent non-physiological tracheal tissue compression have been suggested and even new simple-to-use devices such as a pressure-sensing syringe have been proposed [23-25]. For the most common method of pressure control, the pilot balloon palpation, studies have shown a prevailing inability of clinical staff members to accurately determine the pressure [23,26]. In a more recent investigation, the tendency to overinflate tube cuffs was still dominating, and even more disturbing, this problem was more common in the group of highly experienced anaesthesiologists [27].

In our study, cuff pressures were not controlled at all in nearly half of the cases. In “controlled” cases, the most frequent technique for assessment was measuring using a manometer, followed by insufflating a “specific volume of air”, “palpatory” and “acoustic” methods, whereupon measurements had been performed prior to almost all ICU transfers. Techniques other than direct measurement are unreliable in the prehospital setting.

Acceptable cuff pressures are best achieved by using a manometer.

### Limitations

There are several limitations to this study. During the study period, about half of all mechanically-ventilated, aeromedically-transported patients could not be included (Figure 1). Nearly one quarter of these were excluded to avoid bias, because they were intubated by the study team itself. About one-third were excluded due to patient safety concerns during immediately life-threatening situations during which over-inflation of the cuff is possibly more common. In addition, the cuff pressure was measured using an analogue pressure gauge with awareness of its limited accuracy. We decided to use this routine device with regard to implementation in daily practice. In just one case using the acoustic method, did we evaluate a cuff pressure within the target range. Unfortunately, this method is generally not feasible in the prehospital setting due to noisy environmental conditions. A further limitation is the involvement of several physicians in the study. We did not test interobserver reliability. Furthermore, we did not collect outcome data.

## Conclusions

Tracheal tube cuff pressures in patients intubated prior to aeromedical transport are too high. In our study, cuff pressures were not controlled at all in nearly half of cases. Awareness about this circumstance seems to be absent from daily practice in emergency situations; this seems to apply to anaesthesia personnel, too. We, therefore, recommend the mandatory routine use of a cuff pressure manometer to avoid inappropriately high cuff pressures. Use of a 5 ml syringe may be a perferable pragmatic alternative to the traditional 10 ml syringe. Acknowledging the dangerous increase of cuff pressures during subsequent airborne transport, medical teams responsible for transfer of intubated patients must monitor tracheal tube cuff pressures and adjust the pressures appropriately.

## Competing interests

The authors declare that they have no competing interests.

## Authors’ contributions

FH helped analyse the data and write the manuscript, and performed the statistical analysis. MZ is responsible for the study design, helped analyse the data, and write the manuscript. MB helped collect and analyse the data. WU participated in the study design, helped collect and analyse the data and write the manuscript. All authors read and approved the final manuscript.
